# Music in Mind Training: Producing a theory of change model to evaluate the implementation of an improvisation-based music-making training programme for care home staff working with people with dementia

**DOI:** 10.1177/14713012251319589

**Published:** 2025-02-12

**Authors:** Dougal Henry James McPherson, Robyn Dowlen, Caroline Bithell, Alexander Gagatsis, Alys Young, Lizzie Hoskin, Max Thomas, Cathy Riley, John Keady

**Affiliations:** Creative Manchester, 5292 University of Manchester, UK; School of Allied Health, Social Work and Wellbeing, 6249 Edge Hill University, UK; Martin Harris Centre for Music and Drama, 5292 University of Manchester, UK; Division of Nursing, Midwifery and Social Work, 5292 University of Manchester, UK; Manchester Camerata, UK; Creative Manchester, 5292 University of Manchester, UK; Manchester Camerata, UK; 9022 Greater Manchester Mental Health NHS Foundation Trust, UK; Division of Nursing, Midwifery and Social Work, 5292 University of Manchester, UK; 9022 Greater Manchester Mental Health NHS Foundation Trust, UK

**Keywords:** theory of change, music, improvisation, care staff, care practices, wellbeing, dementia, care home

## Abstract

**Background:**

In the UK, care home staff are often involved in musical practices as part of their professional activities. However, to date there is a lack of relational evidence that underpins improvisational music-making programmes in care homes, as related to the wellbeing of care home staff and musicians who deliver such work. This process evaluation accesses Manchester Camerata’s 20-week ‘Music in Mind Training’ programme for care home staff working with people living with dementia in care homes, with a focus on care home staff.

**Aims:**

(i) To produce a Theory of Change model outlining the core mechanisms of change for Music in Mind Training; (ii) To evaluate the ‘in-the-moment’ and prospective impact of Music in Mind Training on participating care home staff practice and wellbeing.

**Methods:**

Conducted in two care homes, the study employed online observation of hour-long training sessions (*n* = 18), semi structured interviews with participating staff and musicians (*n* = 4), and oral histories interviews with stakeholders involved in programme development (*n* = 18).

**Findings:**

Participating care home staff reported a general increase in their motivation, wellbeing, and confidence through taking part in the training programme, while indicating a drop in confidence related to future delivery at the programme end. The study also indicated how care home staff implemented change to their day-to-day practice by incorporating their learning into interactions with residents in structured music sessions, and more broadly in daily interactions.

**Conclusion:**

The presented Theory of Change model details core interpersonal mechanisms of change for this musical training programme, centred on (1) collaboration, (2) shared values, (3) respect and validation, and (4) openness and reflection, outlining pathways for impact regarding practice change and staff wellbeing. Subject to further refinement and testing, the model could be applied to other contexts to help provide a more rounded account of education and training in dementia care settings.

## Introduction

In the United Kingdom (UK), approximately 70% of older people living in care homes have a diagnosis of dementia (Alzheimer’s Society, 2016), and often with one or more medical comorbidity such as heart failure, chronic obstructive pulmonary disease, and/or diabetes (All-Party Parliamentary Group on Dementia (APPGD), 2016). However, to date, there is very little dementia specific training delivered and sustained within care home environments. That which exists has been shown to improve care home staff’s understandings about the lived experience of dementia, to enable them to feel more attuned to people’s care needs, and to improve feelings of empathy and compassion towards people living with dementia (Surr et al., 2023). The acquisition of this new knowledge by care home staff has been found to be enhanced where access to intensive interventions and ongoing support are provided, and where staff have a dedicated time and space to engage with training activities (Kuske et al., 2007; Surr et al., 2023).

Against this context, the joint Nuffield Trust and King’s Fund report ‘Social Care for Older People: Home Truths’ (Humphries et al., 2016) has revealed the substantial pressures facing the care home sector in the UK and the urgent need to use limited resources more effectively to achieve high-quality care. Some of the key challenges in this area, as also highlighted by the Department of Health and Social Care (2021) report on the adult social care workforce (which includes care home and domiciliary care), revolve around recruiting and retaining staff and maintaining morale and well-being. These findings were reinforced by a recent APPGD (2022) report that acknowledged the pressures in retaining and upskilling the care home workforce, whilst also seeking to deliver personalised care for people living with dementia by improving working practices, leadership, and care home culture. Such pressures were both intensified and exacerbated by the COVID-19 pandemic and the disproportionate number of resident deaths with its concomitant, and legacy, impact on care home staff and family carer wellbeing (Giebel et al., 2022).

While there are significant challenges facing the care home workforce, there are certain working conditions that have been shown to positively impact on some of these key challenges. For example, care home employers/organisations with favourable ‘workforce metrics’, such as opportunities for training and development, have been reported to be more likely to have lower staff turnover and/or higher Care Quality Commission (CQC) score (Skills for Change, 2021). The type of training and development opportunities cover a large body of skills, knowledge and requirements that are necessary for providing a safe, stimulating, and stable care home environment for staff and residents alike.

As part of the provision of a stimulating environment, care home staff, including activities coordinators, hold important positions in enabling access to creative activities for people living with dementia, and other residents, in their day-to-day working practices (Eaton et al., 2023). There are numerous studies which report the positive benefits of engagement with arts programmes, or arts activities, for people living with dementia and care home residents (see: Broome et al., 2019; Curtis et al., 2018; Cutler, 2022). The embedding of arts-based programmes has also been shown to directly impact care staff’s perceptions of work, including work-based satisfaction (Kable, 2015), relationship-building (Hodgson et al., 2023; Windle et al., 2020), and enhancing caring practices through a positive change in attitudes and understandings about the lived experience of dementia. However, these programmes are often delivered by arts organisations, or independent artists, and tend to be short-term and rarely place the upskilling of care home staff in creative activities as central to the programme aims.

There is therefore a pressing need to support care home staff in their everyday work, which includes the performance of tasks that are designed to spark enjoyment, creativity, and wellbeing in all those attending, and to perform this through in-person and/or via remote activities. To address this need, this study focused on understanding and evaluating a live, improvisation-based, music-making training programme – *Music in Mind Training* – implemented within care home contexts. The programme’s facilitators were professional musicians and music therapists, and the participants were care home staff working with people living with dementia in care homes. While people with dementia were beneficiaries of the training from the perspective of Manchester Camerata, they were not participants within this study.

The aims of our study were twofold. First, to produce a Theory of Change model outlining the core mechanisms of change of the Music in Mind Training intervention. Second, to evaluate the ‘in-the-moment’ and prospective impact of Music in Mind Training on participating care home staff practice and wellbeing. The full Theory of Change model will be shared in this paper, and an in-depth examination of the mechanisms of change at the centre of the model will be its primary focus.

## Methods

### What is music in mind training?

Music in Mind Training is an educational development of Manchester Camerata’s Music in Mind programme (https://manchestercamerata.co.uk/community/music-and-dementia/; accessed 15 January 2025), a live music-making programme for people living with dementia based on music-therapy and group improvisation, which has been successfully implemented in community settings and care homes in the North West of England since 2012. To date, Music in Mind has reached over 11,000 people living with dementia and their supporters and has generated similar improvisation-based programmes in Japan, Taiwan, and Sweden. A more complete description of Music in Mind can be found in Dowlen et al. (2022). The training element of Music in Mind, Music in Mind Training, is the focus of this study. Music in Mind Training is a programme for care home staff and is comprised of two educational elements led by professional musicians, in a 20-week pattern of delivery. These are: (i) Weekly provision of face-to-face participatory group music-making sessions for care home residents living with dementia, co-facilitated by professional musicians and care home staff enrolled on the training; and (ii) Fortnightly online reflective training sessions for the care home staff, featuring synchronous and asynchronous learning (virtual meetings with musicians, and independent study using digital resources).

The training is delivered by freelance professional musicians and music therapists contracted by Manchester Camerata. We will refer to these professionals as ‘trainers’ in this paper and care home staff employed at participating homes who take part in the training as ‘staff’, ‘staff carers’ or ‘care home staff’ depending upon context. As the organisational body overseeing the training programme, contracting musicians, and developing pedagogical resources, Manchester Camerata is referred to throughout the paper and the Theory of Change model as the ‘training organisation’.

### Study design and sites

The overall methodological approach of this study was guided by Theory of Change (Astbury & Leeuw, 2010) and operationalised by the UK Medical Research Council (MRC) framework for process evaluation of complex interventions (Skivington et al., 2021). Specifically, the MRC framework refers to a study which ‘aims to understand the functioning of an intervention, by examining implementation, mechanisms of impact, and contextual factors’ (Skivington et al., 2021, p. 8). In the context of this study, the complex (social) intervention is Music in Mind Training and its 20-week programme of activity, whilst the implementation, mechanisms of impact, and contextual factors represent arenas of focus for the presented Theory of Change model.

Music in Mind Training was delivered simultaneously in two care homes across the Northwest of England. The 20-week programme, including 10 training sessions and 10 face-to-face delivery sessions, was undertaken between April and September 2023. Care home sites were identified in collaboration with Manchester Camerata, with approaches being made to care homes who fell within the following inclusion criteria: (i) Medium size so that there was capacity for staff members to take part in the programme; and (ii) Boroughs in which there were reported high levels of social deprivation. Project management of the training programme was led by Manchester Camerata, with the research staff managing the research study embedded within the delivery. Two care homes were recruited into the study:

1. Care home A is a privately-run care home which provides dementia nursing and residential care for a maximum of 120 people. It had an ‘Overall Good’ CQC rating.
2. Care home B is a privately-run, modern care home which provides residential care for a maximum of 70 people. It had an ‘Overall Good’ CQC rating.

Care home staff were eligible for inclusion in the training if they were employed in a caring role (including care workers and activities coordinators) and had the ability to attend the majority of face-to-face delivery sessions (15+) and online reflective sessions (7+).

### Study methods and participants

We used a range of qualitative methods to explore the implementation, mechanisms, and impacts of the training programme for staff wellbeing and practice: (i) online observations of training sessions (*n* = 18, 18 hours); (ii) semi-structured interviews with participant staff (*n* = 2) and trainers (*n* = 4) (3.7 hours); (iii) oral histories interviews with individuals involved in the development of Music in Mind over its 12 year history as at the time of the interviews (*n* = 18, 12.5 hours).

Online observations by DHJM and RD of 18 Music in Mind Training sessions took place on Zoom, across the two participating care homes. Semi-structured interviews with two staff carers took place on Zoom. Staff Carer A was an ‘activities coordinator’ at care home A who attended 9/10 Music in Mind Training sessions alongside involvement in 20 live Music in Mind sessions. Staff Carer B was a ‘wellbeing coordinator’ at care home B who attended 10 Music in Mind Training sessions and 18 live Music in Mind sessions. For staff carers, interviews explored expectations of taking part in the training, key learning and experiences during the training, perceptions of their own wellbeing and job satisfaction, and their perceptions of how the training had influenced their caring practices. Semi-structured interviews with musician trainers took place in person (*n* = 4), and explored their professional backgrounds and experience delivering the training, the support available to them from Manchester Camerata, and their motivations for delivering the training in the short and longer-term. Oral history interviews with Music in Mind stakeholders took place online, and in person. These comprised current and former project managers and senior leadership personnel at Manchester Camerata (*n* = 6), musicians and music therapists contracted by Manchester Camerata (*n* = 8), and researchers from the University of Manchester (*n* = 4). During this part of data collection, interviewees also sketched visual timelines of their involvement with the Music in Mind programme and this was used as an elicitation source during the interview (Marshall, 2019).

Data collection for the study took place between April and December 2023. Anonymised interview transcripts for the staff carers who participated in this study can be accessed via the University of Manchester FigShare repository at the following link: https://doi.org/10.48420/27109360.v1

### Patient and Public Involvement and Engagement (PPIE)

Across the research study, the Open Doors Research Group, a self-advocacy group of people living with dementia based in Salford, Greater Manchester, and facilitated by CR in the authorship, acted in a consultative capacity to shape the direction of the study and to input into the developing Theory of Change model for the training. Six sessions with 14 group members (including a mix of people living with dementia and family carers) were held at regular intervals across the study. In the second meeting, members of the group were visited by a musician and music therapist from Manchester Camerata so that they could ‘experience’ a Music in Mind session for themselves. The groups were co-facilitated by DHJM, RD and CR and, in the last two sessions, members inputted into the language, presentation, and structure of the Theory of Change model.

### Data analysis

Following precedents within Narrative Enquiry in creatively combining analytic methods to engage data from a variety of multi-layered perspectives (Beal et al., 2012; Riessman, 2008), the researchers adopted a syncretic analytic approach drawing upon Reflexive Thematic Analysis (RTA), as outlined by Braun and Clarke (2022), and Narrative Analysis techniques. This approach was grounded in the principle that narratives represent vehicles for sense-making and for personal and collective worlding, by which people ‘shape their daily lives’, articulating ‘who they and others are’ and interpreting their past ‘in terms of these stories’ (Clandinin & Connelly, 2004, p. 375). Aligning with Braun and Clarke’s (2022) model of ‘making the argument’, the analytic approach aimed to present a contextualised and rich ‘tapestry of understanding’ (p. 121) through adopting multiple methods, to illuminate salient emergent aspects of the training programme.

Led by DHJM and RD, members of the research team undertook initial data familiarisation and immersion through reiterative close reading of interview transcripts, examination of oral histories expert timelines (visual event-based documents), field observation notes and transcripts from 18 training sessions. This initial phase was followed by a reiterative and reflexive cycle of inductive coding (within NVivo 12), re-reading, thematic clustering, and reviewing drawing upon Braun and Clarke’s (2006) six stage model of RTA. Within this analytic cycle, three conditions were applied: first, themes were considered relational within the remit of an applied holistic strand of narrative inquiry (Lieblich et al., 1998); second, the performativity and dynamics of narrative telling (i.e., how the narratives were told), were explored in relation to the cultural and social framing of Music in Mind Training and the research study; and third, emergent themes and cluster headings were shared with the Open Doors Research Group who asked critical questions about the presented data.

Recognising the need to make findings accessible to a variety of stakeholder audiences including academic, sector professionals, and the public, the Theory of Change model has been developed in four iterations of graded complexity: (1) complex; (2) streamlined; (3) simplified; and (4) postcard. Language for each iteration has been tailored to different stakeholder groups as previously highlighted, and as the most detailed, the complex Theory of Change model is presented in this paper.

### Ethics

Ethical approval to conduct the study was granted through the University of Manchester Research Ethics Committee (Ref: 2023-15090-27165). Informed consent was obtained from all participants, and was provided in both written and oral (verbal) format.

## Findings

### The theory of change model

**Figure 1.**
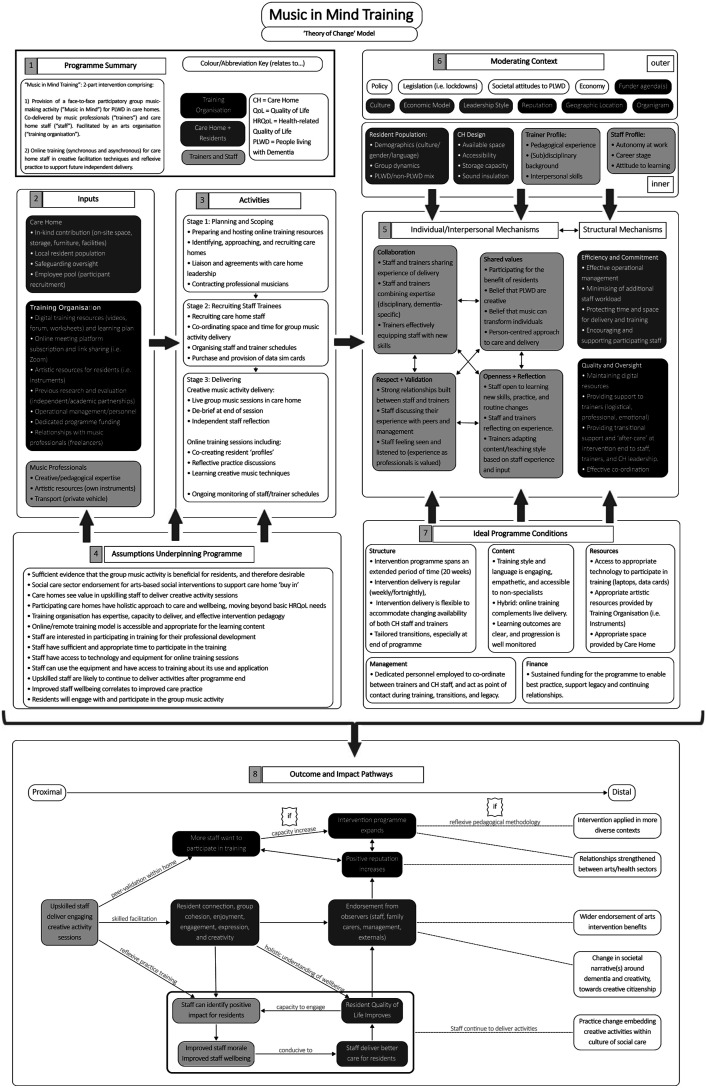
Theory of Change Model (Complex Version) for Music in Mind Training.

Based on analysis of the above data, the complex Theory of Change model is shared in Figure 1 and articulates the core aspects of Music in Mind Training. This model proposes underlying mechanisms of change, moderating contextual factors, and suggests pathways for impact regarding practice change, staff wellbeing, and further implementation of the programme in different contexts.

Whilst the complete, complex model is shared in Figure 1, we have drawn out the summaries of the different boxes within the model to showcase elements of the training programme in turn (e.g., inputs and activities; moderating contextual factors; outcome and impact pathways and so on). This summary is shown in Table 1.

**Table 1.** Summary of boxes within the theory of change model.

Box 1	Programme summary	Replicable description of the intervention and list of abbreviations.
Box 2	Programme inputs	List of programme ‘inputs’ including detail of different stakeholder (i.e., care home, training organisation, music professionals) contributions to the intervention.
Box 3	Programme activities	List of the programme’s core activities involving recruitment, delivery, and ongoing monitoring. Sequential stages of programme planning, resourcing, and implementation.
Box 4	Assumptions Underpinning the Programme	Assumptions which underpin the training programme. It is reasonably assumed that these factors will hold true for the Theory of Change to function as proposed in the model.
Box 5	Mechanisms of Change	Integral processes which facilitate change within the intervention, linking the programme’s activities to proposed outcomes. Detailed across individual/interpersonal and structural (organisational) levels.
Box 6	Moderating Context	Moderating contextual factors impacting the overall implementation of the programme, and in particular the functioning of the mechanisms, identified at inner (personal and logistical) and outer (organisational, social and systemic) levels.
Box 7	Ideal Programme Conditions	Suggested conditions for optimal implementation of the programme’s aims and objectives, based on observation of training sessions, interviews and analysis of oral histories data.
Box 8	Outcome and Impact Pathways	Outcome and impact pathways arranged from ‘proximal’ to ‘distal’, spanning: proposed outcomes most immediately generated by core intervention activity; to prospective impact which is contingent on assumptions of sustained practice (respectively). Proposed outcomes for residents are based on observed benefit reported by staff carers and musicians.

Although we refer to the whole Theory of Change model within this paper, we will specifically focus on the ‘mechanisms of change’ (*see* Figure 1 and Box 5 in Table 1) and the ‘interpersonal mechanisms’ between staff carers and musician-trainers (*see* Figure 1 and Box 7 in Table 1). This is because Box 7 contains propositions for effective implementation of this training programme, based on an analysis of the available data. As a planned outcome of this study, this was also the focus for our ‘best practice recommendations’ made to Manchester Camerata in early 2024 and, in our opinion, would most directly inform the transferability of Music in Mind Training to other practice contexts.

## Mechanisms of change

As Figure 1 reveals, at the centre of the model are six ‘mechanisms of change’, identified at individual-interpersonal and structural levels. While the structural level frames and informs the individual-interpersonal, all mechanisms are considered interactive and reflexive within and across each level. Potential mechanisms at the systemic level have not been explicitly identified in this model; however, the ‘moderating context’ (*see* Figure 1 and Box 6 in Table 1) includes factors which might feasibly be explored from a systemic mechanism perspective; for example, how societal level attitudes to people living with dementia function within the learning environment, or the impact of policy on the planning and implementation of the programme.

Central to the model are the ‘interpersonal mechanisms’, focussed on the personal and pedagogical relationship between staff carers and musician trainers. These are grouped under four main headings but are considered inter-relational and to some extent porous. ‘Collaboration’, for example, can be viewed as an integral aspect of an ‘open and ‘reflective learning environment’, while a ‘person-centred approach’ under the heading ‘Shared Values’ also forms an aspect of musicians’ and carers’ combined disciplinary expertise. The ‘structural mechanisms’ pertain to training programme oversight, co-ordination, organisational resource management, and support for participating professionals. Responsibility for co-ordinating the programme lay with the training organisation (Manchester Camerata), which provided and maintained digital resources and learning content, scheduled training sessions via the Zoom platform, and contracted musicians. In this study, co-ordination was notably reliant on one dedicated part-time staff member at Manchester Camerata who acted as a critical liaison between the musicians, staff carers, care home, and research team for the duration of the programme, as identified by the ‘Ideal Programme Conditions’ (*see* Figure 1 and Box 7 in Table 1). Care home management of staff workload, protected time and space for delivery, and encouragement of participating staff were key structural mechanisms which affected carers’ capacity to participate effectively in the training (i.e., with the greatest depth of learning and potential for subsequent practice change). These mechanisms, in particular, represent an area with scope for refinement and development, as will be addressed below.

### Openness and reflection

Music in Mind’s Training/learning environment was infused with an ethos of open communication and, critically, reflexivity on the part of both trainers and staff-learners. Notwithstanding the challenges and compressed time in which they had to learn, the open attitude of staff carers to learning new skills, and to changes in daily practice and routine, facilitated their engagement with the training materials/discussion and contributed to the establishing of a relationship of trust built with the trainers. Willingness to learn was connected to a motivation, on the part of staff carers, to undertake training ‘for the benefit of the residents’, (see section ‘Shared Values’). The trainers also demonstrated reflexivity in their tailoring of content to the specific staff carers and their care contexts, for the most part effectively altering or modifying more complex musical terminology and reframing it within more accessible language. For example, in one session at care home 2, the trainers discussed the scope to use ‘graphic scores’ - visual prompts used in place of sheet music or notation - as part of musical activities in the home. The trainer explained in lay language the purpose and affordances of a graphic score, highlighting the staff carer had existing artistic skills, which could be repurposed in combination with their developing musical knowledge, for engaging session delivery.

To support effective learning for non-specialists (in music), maintaining an effective bank of accessibly worded and structured resources represents an important mechanism on the part of the training organisation. While a body of digital video resources developed for Music in Mind Training was used at various points during the training we observed, the extent to which staff were expected to engage in asynchronous independent learning was not always apparent. Staff carers reported at times being unable to access the videos directly via the online portal (requiring login), and in general, the researchers observed that their response to the use of training videos was less discursive and engaged than when participating in shared reflective discussions relating to face-to-face music-making.

### Collaboration, respect, and validation

Openness to collaboration, mutual respect and validation - and a recognition of professional expertise in both music and social care - was the foundation from which relationships of trust were built across the course of the programme. The development of this supportive and trusting relationship between trainers and staff was essential for the effective transfer of skills and the smooth running of the programme.

Trust-building was supported through musician-trainers’ regular encouragement and highlighting of carers’ existing skills as social care professionals, while reorienting them towards musical facilitation. Musician-trainers were aware of potential barriers of framing all skills around musical competency, and the potential sense of challenge in facilitating musical activities as non-specialists. Staff carers were therefore encouraged to draw upon their existing knowledge of residents’ day-to-day behaviours and expressions to identify moments of connection and support residents’ sustained engagement in face-to-face delivery. Through reflection on staff’s detailed interpersonal knowledge of residents’ individual communication styles, staff and musicians were able to adapt musical interactions in face-to-face delivery, and identify appropriate means to encourage and support resident participation (e.g., eye contact, touching, supported instrumental playing, talking). While it took some weeks to develop, recognition from all parties of Music in Mind Training as a coming-together of disciplinary/professional expertise softened the hierarchy of the learning environment and facilitated effective learning. Trainers also gave regular positive feedback to staff on their engagement within the training sessions, highlighting in particular their attitude to learning, as the following quote from one musician towards the end of the programme demonstrates:

But I can see I can see the difference in you, [Name], I'm thinking about when we first started this and now, how much confidence you've gained, how much knowledge you've taken on board and you've been so open, and you really absorbed it and you really have been curious about it and interested in it, been brilliant in it.

Sharing of expertise also aided in the development of resident ‘musical profiles’, which formed a substantial aspect of the reflective online sessions. For each resident, musical likes and dislikes, preferences for instruments and musical roles (i.e., leading, following), as well as musical ‘biographies’, were identified based on detailed staff knowledge; this was informed by past staff-resident interactions and information contained in personal care plans. Discussions of cultural demographics raised issues of Eurocentricity, indicating a desire on the part of the professional musicians to diversify the scope and style of music (particularly pre-recorded, non-improvised music) beyond Western and European musical traditions. Different access needs and differences in mobility and dexterity were also discussed at length, such as the physical or structural appropriateness of certain instruments for different residents; staff carers and musicians reflected together on tactility, grip, hold, and playing technique.

However, while ‘profiling’ in this way was an effective activity for learning in the online sessions, the time taken to reflect, and write up such profiles was considered difficult within staff workload. Staff Carer B expressed they always ‘*have a few minutes after the session*’ in which to create profiles, while Staff Carer A emphasised the lack of time available to them, owing to pressures of existing workload inputting similar information into electronic care planning systems (PCS). Trainers’ expectations of staff commitment at times outweighed the time limitations and practicalities of day-to-day working for the staff carers, both of whom worked part-time. Owing to the relationship of trust built with the musicians, however, staff carers openly discussed the challenges of demands on their time within the online training sessions, as the following quote highlights:

I’m just on very tight, like limited timescale. Because after you guys leave, I have to write up every single person who came through the Music in Mind session, and I have to write my own record on an online PCS system, which takes me about 40 [minutes], it literally takes me up until I’m about to leave. (Staff Carer A, Observation)

Efficiency and commitment on the part of the care home in supporting staff engagement, therefore, can be recognised as an important mechanism to enable effective learning. Ensuring protected time and space for Music in Mind’s Training delivery and reflective sessions, while minimising additional staff workload, enables staff engagement with the learning materials and provides time for reflective practice and for implementing skills learned in online training within the context of face-to-face delivery.

### Shared values: ‘Doing it for the residents’

The primary motivation for staff carers’ participation in the training was the perceived benefit for residents:

I also wanted to do it for the residents. That’s why I wanted to do it, to be honest, for the residents. And obviously if it gained my confidence then obviously that’s going to make me happier as a person, in work and out of work. So yeah, then that was a bonus, I guess. (Staff Carer A)

Professional development was also highlighted by Staff Carer B as important to them, but overall, this was secondary to providing activity which would improve resident quality of life. Staff Carer A expressed a desire to undertake training to help residents with *‘their confidence…Help them to make music…Bring them happiness*’, while Staff Carer B identified benefit in the training as *‘something really good that the residents are going to get out of it’*. Staff Carer A’s perception of this benefit was linked to their attitude at work; they expressed that their main workplace motivation was bringing residents happiness ‘*even if it's just for five minutes’*, stating *‘that’s really why I do it’*.

In reflective sessions, this benefit was often narrated in terms of observing identifiable ‘change’ or ‘transformed’ resident engagement, and a narrative of music-as-transformational was frequently co-constructed by both musicians and staff. ‘Unexpected’ musical contributions from residents in face-to-face sessions, their assuming of diverse musical roles (leading, following, singing, call-and-response), their excitement to attend sessions, and degree of tactile play with instruments, were identified as significant indicators of engagement by care home staff and musicians. These were frequently described in terms of narratives of change, as departures from residents’ ordinary behaviours, as the following quote details:

When the music comes on, they change, they change their whole demeanour, they’re happy, they’re singing, they could have been angry and aggressive one minute but then the music comes on and they just change to a different person. So, for me it is rewarding seeing the residents come alive and be happy. (Staff Carer A)

This perception of a positive benefit for residents identified in these moments of departure was therefore ‘rewarding’ for staff carers; a direct link can therefore be drawn between staff carers’ capacity to recognise increased engagement and change as a positive benefit, including in morale (*see* Figure 1 and Box 8 in Table 1). This capacity to recognise engagement was an identifiable, although unwritten, learning outcome of the training. Staff carers were trained to identify the diverse ways in which residents explored sensory (tactile and auditory) interactions with instruments, which, when combined with their existing person-centred practices, allowed them to identify expressions of engagement and enjoyment.

Within the ethos of improvisatory practice, in which all contributions from participants are at the most fundamental level *accepted* (see for example Yamamoto, 2021; Bermant, 2013; i.e., there is no ‘right’ or ‘wrong’ way to contribute) staff were also encouraged to identify moments of engagement (i.e., sounds, expressions, interactions) which were not necessarily predicated on sustained interaction over the course of a whole session, or sequence of sessions, but on their contribution in-the-moment to the improvisation at hand. Focusing on the immediacy and contextuality of interactions, the meaningfulness of these moments of engagement were reflected upon in online sessions between staff and trainers. Musician-trainers also reinforced the importance of facilitating choice and agency for residents, such as their ability to ‘lead’ while staff were encouraged to ‘follow’ or ‘guide’ material in through adaptive, sensitive playing. Staff carers reflected on residents’ interpersonal engagement within the social-musical space of group improvisation (MacDonald & Wilson, 2020), noting moments of connection and interaction with other residents which, like engagement with instruments, were narrated as ‘changed behaviours’ or as ‘different from the norm’.

Consistency of face-to-face delivery was highlighted as a significant facilitator of residents’ engagement in sessions, and lack of consistency was described in negative terms. Care staff reported that they felt regularity of face-to-face delivery, occurring at the same time each week, would allow residents to develop routine. Staff Carer A commented that ‘*whenever there was a bank holiday or something and we missed a week, it was hard to bring it back’*, while Staff Carer B reported that the regularity of sessions gave residents *‘purpose for something to look forward every week’* and allowed them to become familiar with the instruments which were kept in each participating care home.

## Impact for staff participating in Music in Mind Training

### Wellbeing

In interview, both staff carers described their personal wellbeing prior to participating in the training with a moderate positive sentiment (‘*okay*’ and ‘*not too bad*’). Staff Carer B reported some stress in the workplace, attributed to time spent working alone without effective support. When asked directly, Staff Carer A expressed that they felt there had been no significant impact on their wellbeing during the training. However, in response to other (less direct) questions, they expressed an increase in confidence and satisfaction related to participating in face-to-face delivery, and overall stated that the training *‘made them [residents] and myself happier.’* Staff Carer B described their experience of the training as positive, commenting that face-to-face delivery was ‘*something that I and the residents looked forward to’*.

Overall, both staff carer interviews indicated a generally positive impact on personal wellbeing through participating in the training. This was linked closely to the perceived benefit of face-to-face delivery for residents. The developing ability of staff to identify diverse forms of engagement from residents led to an increase in their motivation, improved morale, and sense of job satisfaction. However, they also expressed concern at not being able to sustain a similar standard of delivery following the withdrawal of musicians at the end of the programme. This led to a downturn in confidence in the final weeks of training.

### Practice-change

Staff Carer A believed that residents’ positive experience of face-to-face delivery would enable their subsequent engagement in future sessions. They expressed a desire to sustain delivery in a solo capacity, but indicated that smaller group sizes, and support from other colleagues in the home, would be required owing to the challenge of balancing musical facilitation and attending to residents’ wider care needs. They highlighted the fact that, following the training, they now felt confident in residents’ ability to participate meaningfully in sessions. This led to a desire to run additional sessions, particularly for residents who they perceived as isolated or lonely. They indicated that sustained delivery would require adapting to the context of their home, particularly with regards to staff workload and schedule.

Staff Carer B stated that they had already begun to incorporate aspects of Music in Mind Training into other activities, such as exercise with residents while using instruments. In addition to group sessions, both staff carers had implemented one-on-one music sessions using instruments, and expressed that these activities were ‘more meaningful than before’. They also described being more ‘aware’ of and giving more ‘attention’ to residents, ‘checking in on them’, and understanding different forms of listening and sensory communication, compared to before taking part in the training programme. They felt more knowledgeable about different instruments and their properties and indicated their intention to purchase more instruments for the home to use in future delivery.

Both staff carers therefore indicated an element of practice change, whereby music-making was more embedded as part of their care practice than before taking part in the programme. However, both also highlighted practical challenges in implementing this change within their respective care contexts.

## Discussion

In this article, we have outlined central aspects of our complex Theory of Change model, focussing on staff carer experience within Music in Mind Training. We have highlighted the interpersonal mechanisms central to the learning environment, foregrounding the relationship between staff carers and musician-trainers, with reference to structural mechanisms (programme oversight and structure). We have detailed how participating staff carers perceived a general increase in their motivation and confidence through taking part in this programme, while also indicating a drop in confidence related to future delivery at the programme end. We have detailed how staff carers implemented change to their day-to-day practice by incorporating aspects of Music in Mind methods into their interactions with residents in structured sessions and more broadly in daily interactions, but noting the need for adaptation around availability and resources within different care home contexts in the future.

Whilst these are important developments, we need to highlight that the mechanisms of change identified within the Theory of Change model are contingent on the specific pattern of planning, delivery, and development of this iteration of Music in Mind Training. While we have proposed factors (*see* Box 7 in Table 1) which concern the replicability and transferability of this type of programme, alternative programme designs will undoubtedly yield different challenges and structural considerations, not least concerning access to resources, scheduling and staff availability, and different resident demographics (*see* Box 6 in Table 1).

Based on our observations of training sessions, future consideration for effective replication of the programme would be a more focussed oversight on the style of digital resources, their usage, and the expectations of staff in engaging with them. In our experience, care home staff would benefit from more direct explication of resource use, with clear learning outcomes and expectations detailed from the outset. However in general, the reflexivity and openness on the part of staff carers and trainers in this programme afforded a flexible and adaptive pedagogical environment; content and delivery style were reframed contingent on the needs and experience of the participating professionals, contributing to the relationship of trust. This flexibility represents a skill of this programme’s facilitators, and a strength within its design.

Analysis of staff and musicians’ reflective sessions also points to the effectiveness of ‘in-the-moment’ practices, such as improvised music-making, as vectors for engagement, enjoyment, and improvement of holistic quality of life for people living with dementia in care contexts (see also Dowlen et al., 2022; Dowlen & Keady, 2024; Keady et al., 2022; Pavlicevic et al., 2015). The data suggests value in equipping care home staff with the confidence and skills to enable moment-to-moment connection in activity sessions (whether arts-based, or otherwise), and more broadly in day-to-day practice. Through taking part in the programme, staff became more skilled in identifying engagement with their residents through improvised music-making, we observed improvements in staff’s self-reported sense of wellbeing, as well as musicians’ sense of fulfilment through delivery of Music in Mind in their role as trainers. This indicates potential for such programmes to have a positive impact on trainers, as much as on learners.

Within this programme, the building of ‘musical profiles’ for residents – encompassing not only their likes and dislikes, but their favoured interactions with instruments and contributions to live performing – represents a form of person-centred practice which directly acknowledges the creative capacities of people living with dementia, reflecting upon their meaningful contribution to improvised music sessions. The moment-focused space of improvising represents a unique environment for residents living with dementia in that it is neither reminiscence-focussed nor future-projecting; rather, it focuses on meaningful, co-creative live interaction across both social and musical domains. That said, how to measure the immediate and ripple effects of such in-the-moment experiences remains both a methodological and ethical challenge.

Finally, the interdisciplinary of the research team was invaluable in addressing the complexity of this social intervention. Our team brought an understanding of applied musical practice, combined with social care, PPIE, health sciences, broadcasting and musicological expertise, to generate rich discussion during the lifetime of the study. This was particularly focussed around the accessibility of the training resources, the practicalities of day-to-day working for care home staff, the importance of specific instruments, facilitatory methodologies to empower residents, and the need for structural support for all parties to ensure the most effective learning environment conducive to positive impact. This coming together of expertise provided a valuable collaborative lens through which to interrogate data from social and practice-centred perspectives. Going forward, we would suggest that attention to describing and reporting interdisciplinary team practices will become vital in framing such research endeavour in dementia studies.

## Study limitations

Our study has three main limitations. First, whilst the dataset includes a significant amount of rich qualitative data across observations, interviews, and field notes, we consider this Theory of Change model to be propositional in the sense that the sample size of participating staff and care homes was small. This was owing to significant challenges in recruitment, in part precipitated by the commencement of this study at the tail end of the COVID-19 pandemic, which limited uptake to the training. Second, the focus of our study was on care home staff and musicians, and whilst the PPIE group were closely involved in the analysis of data, further work and development of the Theory of Change model would benefit directly from the embedded involvement and expertise of people living with dementia. Third, to better represent the care home sector, future research should adopt a longitudinal or multiple case-study design to track proposed impact pathways and their contingencies over an extended period of time and with a greater sample size.

## Conclusion

In this article we have presented the complex Theory of Change model for the Music in Mind Training programme. We have detailed the interpersonal mechanisms central to the working of the programme, and highlighted both affordances and challenges in the social intervention’s implementation and implications for the transferability of the intervention into future contexts. We have outlined the impact on participating staff, their reports on wellbeing and implications for practice-change, as indicated by our qualitative interview and observation data. To enhance reporting and authenticity, future work should draw together the voices of staff carers and residents living with dementia using creative research methods as a space for reflection and discussion (Kara, 2020).
